# Editorial

**DOI:** 10.1080/26415275.2020.1724646

**Published:** 2020-03-30

**Authors:** Anne Peutzfeldt, Jon E. Dahl

**Affiliations:** a Institute of Odontology, Copenhagen, Denmark; b Nordic Institute of Dental Materials, Oslo, Norway

Dear Reader,

Biomaterial Investigations in Dentistry (BIiD) was launched in mid-2014 under its former name, *Acta Biomaterialia Odontologica Scandinavica*, as one of the first international, open access journals within dentistry.

Open access publishing not only gives all ‘stakeholders’ free access to the research that they have, directly or indirectly, helped fund, but it also maximizes the impact of the research, while leaving the copyright with the authors.

BIiD is owned by the non-for-profit organization Acta Odontologica Scandinavica Society and is a member of the Committee for Publication Ethics (COPE) and it follows the COPE guidelines. BIiD is indexed in PubMedCentral and therefore searchable in PubMed. Furthermore, the journal is included in the Open Access registry DOAJ and, as of this year in Publons, a reviewer recognition platform.

We, the editors, work dedicatedly toward BIiD qualifying for an impact factor. Together with our international team of research-active editorial board members, we also strive arduously to ensure that all submitted manuscripts undergo an unbiased and impeccable peer review process, an approach that we believe is essential when striving to establish BIiD as a leading international and high-quality journal in the field of dental biomaterials.

